# Real-world experience with a Paclitaxel-Coated Balloon for the treatment of atherosclerotic infrainguinal arteries: 12-month interim results of the BIOLUX P-III registry first year of enrolment

**DOI:** 10.1590/1677-5449.007317

**Published:** 2017

**Authors:** Marianne Brodmann, Thomas Zeller, Johnny Christensen, Christoph Binkert, Lubomir Spak, Henrik Schröder, Paolo Righini, Giovanni Nano, Gunnar Tepe

**Affiliations:** 1 Medical University Graz, Department of Angiology, Graz, Austria.; 2 Universitaets-Herzzentrum Freiburg-Bad Krozingen, Department of Angiology, Freiburg, Bad Krozingen, Germany.; 3 Kolding Hospital, Department of Radiology, Kolding, Denmark.; 4 Institut für Radiologie, Kantonsspital Winterthur, Winterthur, Switzerland.; 5 East Slovak Institute of Cardiovascular Diseases, Department of Cardiology, Kosice, Slovakia.; 6 Jewish Hospital, Center for Diagnostic Radiology and Minimally Invasive Therapy, Berlin, Germany.; 7 IRCCS Policlinico San Donato, 1st Vascular Surgery Department, San Donato Milanese, Milan, Italy.; 8 Institute for Diagnostic and Interventional Radiology, Rosenheim Hospital, Rosenheim, Germany.

**Keywords:** Peripheral artery disease, Paclitaxel-Coated Balloon, endovascular therapy, doença arterial periférica, balão revestido com paclitaxel, terapia endovascular

## Abstract

**Background:**

Endovascular management of atherosclerotic infrainguinal arteries recently shifted towards drug eluting devices, designed to locally prevent the restenosis process. Numerous clinical studies report an advantage of drug coated balloons over uncoated balloon angioplasty in treating lower extremity peripheral artery disease. However, as coating and balloon platforms are different, each device requires dedicated clinical evaluations.

**Objective:**

The aim of the study is to further investigate the safety and effectiveness of a Paclitaxel-Coated Balloon for the treatment of atherosclerotic infrainguinal arteries in a real-world setting.

**Methods:**

203 patients out of a final sample of 882 were enrolled in this prospective multicenter, observational, all-comers registry during the first 12 months. The primary endpoints were major adverse events (defined as procedure or device related death within 30 days post index procedure, clinically-driven target lesion revascularization or major target limb amputation) at 6 months and freedom from clinically-driven target lesion revascularization at 12 months. Both endpoints were adjudicated by a Clinical Events Committee.

**Results:**

Mean patient age was 70.2±10.4 years (60.1% male). 47.3% of the patients were diabetic and 67.5% had a history of smoking. Severe claudication was reported in 37.4% and 40% had critical limb ischemia. 257 lesions, including 13.2% in the infrapopliteal territory, were treated with Passeo-18 Lux (mean lesion length 75.1 mm±69.4, 20% occlusions, 76.3% calcified). At 6 months, the rate of major adverse events was 5.5% (95%CI 3.1-9.7). Freedom from clinically-driven target lesion revascularization at 12 months was 93.2% (95%CI 89.1-95.8). All causes mortality was 6.5% (95%CI 3.8-11.0) and overall amputation rate was 4.2% (95%CI 2.1-8.3) at 12 months.

**Conclusion:**

In a real-world environment, the BIOLUX P-III registry preliminary results confirm the safety and efficacy of the Paclitaxel-Coated Passeo-18 Lux balloon as a stand-alone treatment option for atherosclerotic infrainguinal arteries.

## Resumo

**Contexto:**

O manejo endovascular de artérias infrainguinais ateroscleróticas recentemente tem mudado para dispositivos farmacológicos, desenhados para impedir localmente o processo de reestenose. Numerosos estudos clínicos descrevem uma vantagem da angioplastia com uso de balões farmacológicos sobre os balões convencionais no tratamento de doença arterial periférica dos membros inferiores. No entanto, considerando que as plataformas do revestimento farmacológico e dos balões são diferentes, cada dispositivo requer avaliações clínicas específicas.

**Objetivo:**

Fazer investigação adicional sobre a segurança e eficácia de um balão revestido com paclitaxel para o tratamento de artérias infrainguinais ateroscleróticas em um cenário de mundo real.

**Métodos:**

203 pacientes de uma amostra final de 882 pacientes foram incluídos neste registro prospectivo observacional multicêntrico de inclusão sequencial, durante os primeiros 12 meses. Os desfechos primários foram eventos adversos maiores (definidos como morte relacionada ao procedimento ou ao dispositivo em até 30 dias após o procedimento-índice, necessidade de revascularização da lesão-alvo ou amputação significativa do membro-alvo) em 6 meses e ausência de de revascularização da lesão-alvo em 12 meses. Ambos os desfechos foram adjudicados por um comitê de eventos clínicos.

**Resultados:**

A idade média foi 70,2±10,4 anos (60,1% sexo masculino). 47,3% dos pacientes eram diabéticos, e 67,5% tinham história de tabagismo. Claudicação severa foi relatada em 37,4%, e 40% apresentava isquemia crítica de membro. 257 lesões, incluindo 13,2% em território infrapoplíteo, foram tratadas com o balão Passeo-18 Lux (comprimento médio das lesões 75,1 mm±69,4, 20% oclusões, 76,3% calcificadas). Aos 6 meses, a taxa de eventos adversos maiores foi de 5,5% (95%CI 3,1-9,7). A ausência de revascularização da lesão-alvo aos 12 meses foi de 93,2% (95%CI 89,1-95,8). Mortalidade por todas as causas foi de 6,5% (95%CI 3,8-11,0) e a taxa geral de amputação foi de 4,2% (95%CI 2,1-8,3) aos 12 meses.

**Conclusão:**

Em um cenário de mundo real, os resultados preliminares do registro BIOLUX P-III confirmam a segurança e eficácia do balão revestido com paclitaxel Passeo-18 Lux como opção de tratamento único para artérias infrainguinais ateroscleróticas.

## INTRODUCTION

Peripheral artery disease (PAD) is a global disease affecting more than 200 million patients worldwide. From 2000 to 2010, the number of patients with PAD increased by almost a quarter, and the rate of increase in low or middle-income countries was even greater (28.7%), because of the rise in life expectancy in those populations.^1^

PAD is caused by atherosclerotic plaques that result in stenosis or occlusion of major arteries supplying the lower extremities. Obstruction of lower limb arteries results in several clinical presentations ranging from no symptoms, through life-style limiting intermittent claudication (IC), in which patients’ walking ability is impaired, to critical limb ischemia (CLI), in which arterial flow is so severely reduced it causes chronic ischemic rest pain or even ischemic skin lesions, such as ulcers or gangrene.^1^^,^ 2

Management of PAD includes mitigation of risk factors (smoking cessation, exercise therapy…), medication, and ultimately limb revascularization procedures.^2^ For a long time, surgery has been considered the gold standard treatment for revascularization. However, since the late 1990s, with the introduction of new endovascular devices, endovascular treatment has become the first line therapy for IC and CLI patients.

Percutaneous transluminal angioplasty (PTA) or balloon angioplasty is often the initial step in an endovascular approach to infrainguinal arterial obstructive disease. This technique has been proven to immediately restore blood flow safely and effectively. However, the vascular inflammation injury resulting from the inflated balloon (barotrauma) entails hypertrophic neointimal formation through vascular smooth muscle cell (VSMC) proliferation and constrictive vascular remodeling.^3^ As a consequence, post-PTA restenosis rates are relatively high with 12-month primary patency rates of around 52% in well-selected lesions from randomized clinical studies.^4^^,^ 5

Stents offer a mechanical scaffold that prevents vessel recoil and negative remodeling after PTA. However, stent fractures and the presence of a permanent implant hindering the vessel’s natural motion prolong vessel inflammation and subsequent neointimal proliferation within the stent struts. In a recent review, Aghel et al reported 12 month primary patency slightly exceeding 80% for nitinol self-expanding stents and drug eluting stents used in moderate-length superficial femoral artery (SFA) stenosis.^6^

Drug coated balloons (DCB) combine balloon angioplasty and the release of an anti-proliferative drug into the vessel wall during balloon inflation. Unlike stenting, DCBs allow the vessel to retain its mechanical properties and prevent the restenosis caused by barotrauma. In addition, since a permanent scaffold is not left behind, all future endovascular and surgical intervention options remain available.

Paclitaxel is an antimitotic drug. Its lipophilic properties enable rapid absorption by the vessel wall upon contact with the coated balloon surface. All DCBs currently on the market use paclitaxel as anti-proliferative agent in their coating. However, each DCB on the market is unique in terms of paclitaxel load (2 to 3.5 µg/mm), excipient, balloon material and coating technology.

Data from randomized controlled trials revealed 12 month primary patency ranging from 65 to 82.2%.^4^^,^ 5 ^,^ 7 A recently published meta-analysis of 11 randomized controlled trials demonstrated an advantage of DCB compared to uncoated balloon angioplasty in treating lower extremity peripheral artery disease, as indicated by improved primary vessel patency, and late lumen loss for up to two years and target lesion revascularization and binary restenosis rates for up to five years.^8^ However, it is well known that the coating technology plays a major role in the DCB performance. In particular, the excipient has to maintain the coating integrity during balloon tracking to the target lesion, while allowing the release of a therapeutic dose of the drug to the vessel upon inflation and contact with the vessel wall.

The Passeo-18 Lux DCB (Biotronik AG, Buelach, Switzerland) obtained CE-marking in Europe in January 2014. It combines the Passeo-18 PTA balloon catheter with a coating matrix composed of paclitaxel (3 mcg/mm^2^) and butyryl-tri-n-hexyl citrate (BTHC) as an inert excipient.

The safety and efficacy of the Passeo-18 Lux DCB for the treatment of PAD have been investigated in two first -in-man randomized (1:1) controlled studies,^9^^,^ 10 Passeo-18 PTA versus Passeo-18 Lux. The BIOLUX P-I trial^9^ and BIOLUX P-II trial^10^ results report safety and efficacy of the Passeo-18 Lux DCB for the treatment of atherosclerotic lesions in respectively, femoropopliteal and infrapopliteal arteries. Results are consistent with previously reported DCB trials with the same anti-proliferative drug.

The purpose of the BIOLUX P-III all comers global registry (ClinicalTrials.gov; identifier: NCT02276313) is to confirm the first-in-man data of Passeo-18 Lux DCB in a real-world population of patients with obstructive disease of the infrainguinal arteries.

In this article, we will present the 12-month results for subjects included during the first year of enrolment.

## METHODS

### Study design and study population

BIOLUX P-III is a prospective, international, multicenter, all comers registry aiming to confirm the safety and effectiveness of the Passeo-18 Lux drug-coated balloon for treatment of atherosclerotic disease in the infrainguinal arteries in daily clinical practice. Furthermore, analysis of dedicated pre-defined patient subgroups, including, inter alia, diabetes, complex TASC C & D lesions and infrapopliteal lesions are planned.

All patients with lesion(s) in the infrainguinal arteries suitable for endovascular therapy treated with, or scheduled to be treated with, the Passeo-18 Lux DCB qualify for the registry. At least 700 patients were to be enrolled. The cohort has been extended to 882 patients to reach pre-specified subgroups sizes. Main exclusion criteria are failure to successfully cross the target lesion with a guide wire (successful crossing means tip of the guide wire distal to the target lesion) and absence of flow limiting dissections or perforations prior to DCB treatment.

Patients underwent percutaneous intervention with the Passeo-18 Lux DCB as per its instructions for use, according to standard of care and the investigator’s standard clinical practice. Therefore, use of adjunctive endovascular devices or techniques (cutting balloon, atherectomy, stents) was not restricted. The numbers of lesions treated with the Passeo-18 Lux was at the investigator’s discretion and according to the clinical practice at the site.

The study, which was approved by the respective independent ethical committees, is conducted according the Declaration of Helsinki, as well as parts of International Conference on Harmonization Good Clinical Practices (ICH-GCP), and ISO 14155 applicable to registries. All patients provided written informed consent.

Monitoring and data analysis is organized by the trial’s sponsor, Biotronik AG (Buelach, Switzerland). The trial is registered on the National Institutes of Health website (ClinicalTrials.gov; identifier NCT02276313).

### Study devices

The Passeo-18 Lux DCB, CE-marked in Europe since January 2014, is identical to the commercially available Passeo-18 PTA balloon catheter with the addition of a homogeneous coating of paclitaxel as the lipophilic anti-proliferative substance (3 μg of paclitaxel per mm^2^ balloon surface). The paclitaxel is incorporated in a delivery matrix of the excipient BTHC, which binds the drug into a microcrystalline structure to improve uptake into the vessel wall. BTHC degrades to citric acid and alcohol in the body. The balloons are available in diameters of 2.5 to 7.0 mm and lengths of 40 to 120 mm.

### Endpoints and definitions

The primary endpoints are freedom from Major Adverse Events (MAE) within 6 months post-index procedure and freedom from clinically driven target lesion revascularization (CD TLR, defined as any re-intervention performed for ≥ 50% diameter stenosis [visual estimate] at the target lesion after documentation of recurrent clinical symptoms of the patient) within 12 months post-index procedure.

All MAE and CD TLR were adjudicated by an independent Clinical Events Committee (CEC).

Secondary endpoints are freedom from MAE at 12 and 24 months; freedom from CD TLR at 6 and 24 months; freedom from CD target vessel revascularization at 6, 12, and 24 months; primary patency at 12 and 24 months; change in mean Ankle Brachial Index (ABI) at 6, 12, and 24 months (and prior to re-intervention); change in Rutherford classification at 6, 12, and 24 months (and prior to any re-intervention) compared to the pre-procedure Rutherford classification; rates of freedom from major and minor target limb amputation at 6, 12, and 24 months and overall amputation rate; Patient-reported outcomes assessment: Pain scale (Wong-Baker Face®) and Walking Impairment Questionnaire (WIQ) at 6, 12, and 24 months, compared to the pre-procedure score; device success; technical success; procedural success.

For baseline lesion length, consecutive multiple single lesions with a healthy segment ≤ 2 cm between them were considered as one lesion.

MAE are defined as composite of device- and procedure-related mortality through 30 days, major target limb amputation, and clinically-driven target lesion revascularization. Major amputations were above the ankle.

Primary patency was defined as freedom from > 50% restenosis in the target lesion as indicated by a duplex ultrasound peak systolic velocity ratio (PSVR) > 2.5 or by visual assessment of an angiogram with no clinically-driven re-intervention.

Technical success was defined as successful completion of the endovascular procedure and immediate morphological success with ≤ 50% residual diameter reduction of the treated lesion as determined by visual estimation. Device success was defined as successful delivery, inflation, deflation, and retrieval of the Passeo-18 Lux DCB. Procedural success was defined as technical and device success with no occurrence of any MAE during the hospital stay.

### Statistical analysis

Endpoint analysis was performed on data available from patients recruited during the first year of enrolment. Considering the observational registry design, BIOLUX P-III does not involve a hypothesis-driven sample size estimation. However, assuming a primary outcome freedom from CD TLR rate of 90% at 12 months, a sample size larger than 150 subjects will provide an acceptable error in the estimate of ± 5% allowing sufficient precision for interim- and sub-group analysis.

For quantitative variables, the mean values, standard deviation, maximum and minimum were determined. A two-sided 95% confidence interval (95%CI) for the mean was calculated when relevant. For qualitative variables, absolute and relative frequencies were determined. For proportions, exact Binomial two-sided 95%CI was calculated when relevant. The rate of freedom from CD TLR and MAE and its individual components were estimated using the Kaplan-Meier method. Estimates are presented with the 95%CI. The chi-square or Fisher’s exact tests were used to compare binary variables, and Student’s *t* test or the Wilcoxon-Mann-Whitney test were used to compare continuous variables for independent samples. Follow-up comparisons were performed using Wilcoxon-signed-rank tests. Standard error was calculated using Greenwood’s formula. Statistical calculations were performed using SAS software (version 9.1.3; SAS Institute, Inc, Cary, NC, USA).

## RESULTS

### Patient population

**Figure 1:**
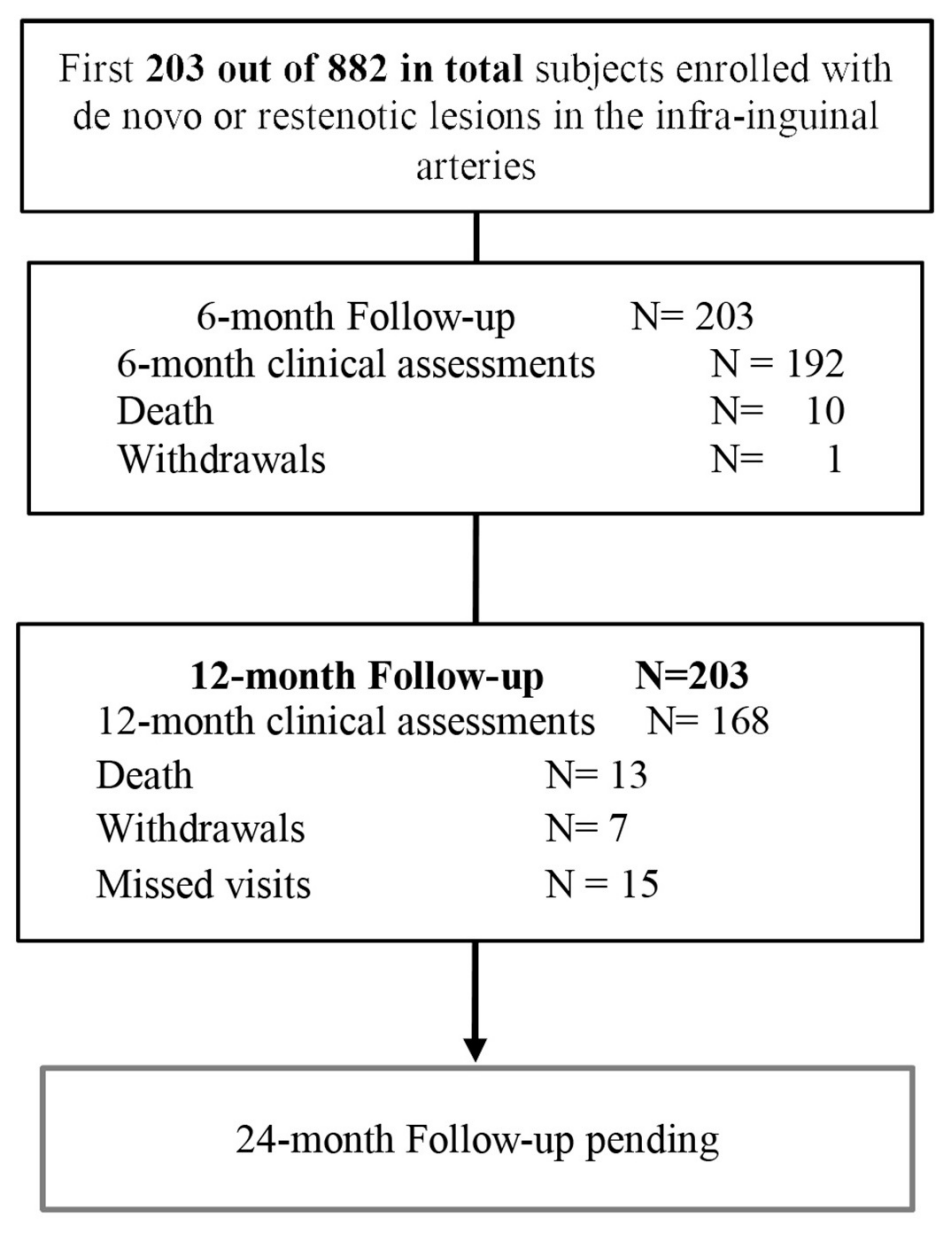
Patients flow chart.

Between October 2014 and September 2015, 203 patients (122 men) out of 882 for the full cohort; mean age 70.2±10.4 years with 257 lesions were treated with Passeo-18 Lux at 20 centers worldwide (Table 1, Figure 1).

**Table 1:** Enrolment per country.

**Countries**	**# Subjects**
Austria	72
Germany	54
Denmark	15
Slovakia	12
Switzerland	12
Belgium	11
Spain	9
Italy	8
Netherlands	6
France	2
Singapore	2

Almost half of the patients (47.3%, 96 patients) were diabetic and two-thirds (67.5%, 138 patients) had a history of smoking. Severe claudication was reported in 37.4% (76 patients) with 40% (81 subjects) CLI (Rutherford class > 3). Demographics and comorbidities are summarized in Table 2.

**Table 2:** Baseline patient demographics.

**Total = 203**	**n (%), if not indicated otherwise**
Age, yrs (mean ± SD), [Min; Max]	70.2±10.4 [44.0; 94.0]
Male	122 (60.1%)
Hypertension	175 (86.2%)
Hyperlipidemia	143 (70.4%)
Smoking	137 (67.5%)
Current Smokers	52 (38.0%)
History of PAOD	120 (59.1%)
Previous PVI/Surgeries	115 (56.7%)
Diabetes	96 (47.3%)
Coronary artery disease	80 (39.4%)
Cerebrovascular disease	49 (24.1%)
Rutherford classification (n = 203)	n (%)
1-mild claudication	1 (0.5%)
2-moderate claudication	27 (13.3%)
3-severe claudication	76 (37.4%)
4-ischemic rest pain	18 (8.9%)
5-minor tissue loss	47 (23.2%)
6-major tissue loss	16 (7.9%)
Unknown	18 (8.9%)
ABI (n = 129 limbs) (mean ± SD)	0.7±0.2

### Procedure and lesion details

Target lesion baseline characteristics and procedure details are summarized in Table 2. The average lesion length was 75.1 mm±69.4 and 20% of lesions were occluded.

The majority of lesions were located in the superficial femoral artery (56.4%, 145/257), almost a quarter (23%, 59/257) in the popliteal artery, and 13.2% (34/257) were in the infrapopliteal territory. More than three-quarters of the lesions were calcified, including almost 12% that were heavily calcified. Device success was 98.5% and technical success was achieved in 98.4%. Procedural success was 95.6% and 13.2% of the lesions required at least one stent.

Procedure and lesion details are presented in Tables 3 and 4.

**Table 3:** Lesion characteristics.

**n = 257 lesions**	
Lesion Length, mm (mean ± SD)	75.1±69.4
Lesion Length distribution	
≤ 100 mm	202 (78.6%)
> 100 mm and ≤ 150 mm	27 (10.5%)
> 150 mm and ≤ 200 mm	12 (4.7%)
> 200 mm	15 (5.8%)
Unknown	1 (0.4%)
Reference Vessel Diameter, mm (mean ± SD)	4.7±1.0
Extent of stenosis (%)	86.3±13.1
TASC Classification	
A	113 (44%)
B	88 (34.2%)
C	33 (12.8%)
D	20 (7.8%)
Unknown	3 (1.2)
Calcification	
None	60 (23.3%)
Mild/Moderate	166 (64.6%)
Heavily calcified	30 (11.7%)
Unknown	1 (0.4)
De Novo Lesion (n, %)	135 (52.5%)
Re-Stenosis (n, %)	37 (14.4%)
In Stent Restenosis (n, %)	31 (12.1%)
Occlusion (n, %)	54 (21.0%)
Common Femoral	1 (0.4%)
Superficial Femoral Artery	145 (56.4%)
Femoropopliteal	9 (3.5%)
Popliteal Artery	59 (23.0%)
Tibioperoneal Trunk	9 (3.5%)
Anterior Tibial Artery	13 (5.1%)
Posterior Tibial Artery	3 (1.2%)
Peroneal Artery	3 (1.2%)
Combination of Infrapopliteal Arteries	6 (2.3%)
Other (Bypass, Iliac)	9 (3.5%)

**Table 4:** Procedure details.

Device success (n = 326 devices)	98.5% (321/326)
Technical success (n = 257 lesions)	98.4% (253/257)
Vessel preparation	70.8% (182/257)
POBA	63.0% (162/257)
Cutting/scoring balloon	4.7% (12/257)
Rotational thrombectomy	2.3% (6/257)
Atherectomy	0.8% (2/257)
Combination DCB + Stents	13.2% (34/257)
Procedural success (n = 203)	95.6% (194/203)

Device success: successful delivery, inflation, deflation, and retrieval of the Passeo-18 Lux DCB. Technical success: successful completion of the endovascular procedure and immediate morphological success with ≤ 50% residual diameter reduction of the treated lesion as determined by visual estimation. Procedural success: technical and device success without the occurrence of any MAE during the hospital stay.

### Primary endpoints

**Figure 2:**
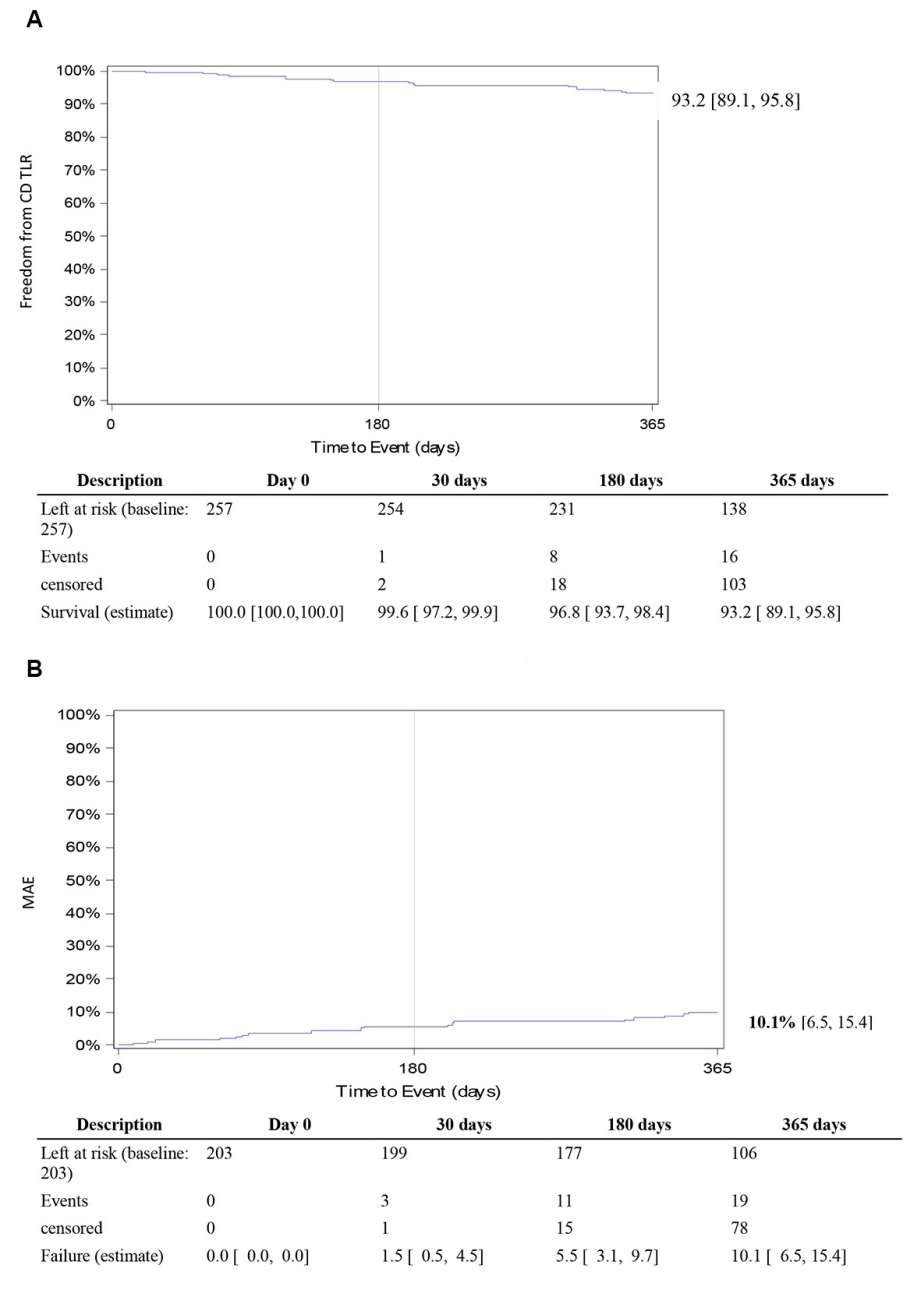
(A) Twelve-month freedom from clinically-driven target lesion revascularization; (B) Twelve-month Major Adverse Event Rate.

The CEC adjudicated MAE rate was 5.5% (95%CI 3.1-9.7) at 6 months (180 days), and 10.1% (95%CI 6.5-15.4) at 12 months (365 days). Freedom from clinically-driven TLR at 12 months, as adjudicated by the CEC, was 93.2% (95%CI 89.1-95.8) (Figure 2). Only one patient died from an unknown cause within 30 days post index procedure. All-causes mortality rate was 6.5% (95%CI 3.8-11.0) at 12 months. Two major amputations (1.0% [95%CI 0.2-3.9]) occurred at 18 and 75 days post index procedure in patients with baseline Rutherford categories of 6 and 5. Overall amputation rate, including minor amputations, was 4.2% (95%CI 2.1-8.3) at 12 months. Safety and effectiveness outcomes are detailed in Table 5 and Figure 2.

**Table 5:** Key outcomes.

	**6 months**	**12 months**
MAE^*^	5.5% (11)	10.1% (19)
Death	1	1
CD TLR	8	16
Major amputation	2	2
CD TLR	8	16
All causes of death	10	13
Amputations (All)^†^	6	8
Major amputations	2	2
Minor amputations	5	7
Change in ABI/TBI (mean ± SD)	0.23±0.30	0.17±0.26
Change in Rutherford classification (mean ± SD)	-2.39±1.74	-2.45±1.90

* Data are given as the Kaplan-Meier estimated percentage;

† First event in time.

### Secondary endpoints

**Figure 3:**
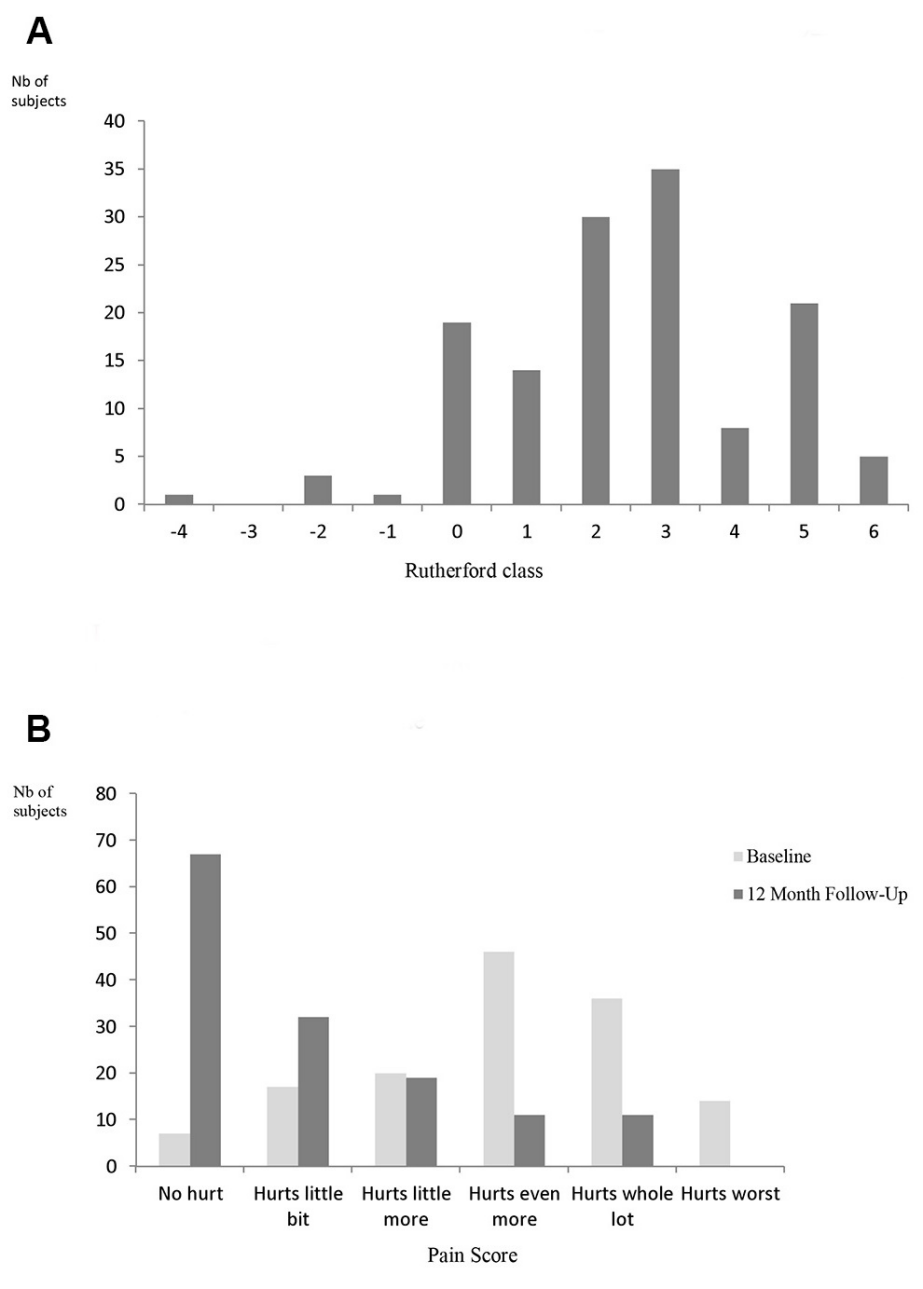
Change in Rutherford classification (12 months vs. baseline) – paired data (n = 137 subjects). (A) Negative figures correspond to Rutherford classification improvement. For instance “-3” correspond to an improvement from RC 4 at baseline to RC 1 at 12 months. (B) Change in Pain Scale (12 months vs baseline).

Primary patency was 85.4% (95%CI 80.2-89.4) at 12 months. Change in ABI at 12 month compared to baseline was 0.17±0.26 (p < 0.001). The vast majority of 82.7% of patients improved significantly by at least one Rutherford category at 12 months compared to baseline whereas 75.4% improved significantly according to the Pain Scale (Figure 3).

Key secondary outcomes details are included in Table 5.

## DISCUSSION

BIOLUX P-III is the first all-comers registry to report results for endovascular treatment of atherosclerotic infrainguinal arteries including tibial arteries with a drug-coated balloon, in a real-word setting including patients suffering from PAD at Rutherford categories 5 and 6. Published data from other global registries^11^^-^ 13 are limited to femoropopliteal arteries and patients with Rutherford classes 1 to 4.

In the BIOLUX P-I trial,^9^ a prospective, international, multicenter, randomized (1:1) controlled trial, 60 patients with stenosis or occlusion of the superficial femoral artery (SFA) and popliteal arteries were treated with either Passeo-18 PTA or Passeo-18 Lux DCB. At 6 months, patients treated with the Passeo-18 Lux DCB had a significantly lower late lumen loss (0.51±0.72 vs. 1.04±1.00 mm, p = 0.033) and binary restenosis (11.5% vs. 34.6%, p = 0.048) than the control PTA group, respectively. There were no deaths and 1 minor amputation was observed in the DCB group compared with 2 deaths and 2 minor amputations in the PTA group. No major amputations or thrombosis occurred in either group.

In the BIOLUX P-II trial,^10^ a prospective, international, multicenter, randomized (1:1) controlled first-in-man study, 72 patients suffering from claudication or critical limb ischemia, and stenosis, restenosis or occlusion of the infrapopliteal arteries were treated with Passeo-18 PTA or Passeo-18 Lux DCB. The primary safety endpoint, rate of major adverse events (MAE), (a composite of all-cause mortality, target extremity major amputation, target lesion thrombosis, and target vessel revascularization at 30 days), was 0% in the Passeo-18 Lux DCB group versus 8.3% in the PTA group (p = 0.239). Patency loss at 6 months, was 17.1% in the DCB group versus 26.1% in the PTA group (p = 0.298). Major amputations of the target extremity occurred in 3.3% in the Passeo-18 Lux DCB group versus 5.6% in the PTA group at 12 months.

The BIOLUX P-III all-comers registry interim 12-month results confirm Passeo-18 Lux safety and efficacy. Only two major amputations occurred in patients with baseline Rutherford categories of 6 and 5. At 12 months, freedom from clinically-driven TLR was 93.2% and the MAE rate was 10.1%. These outcomes are comparable to previous Passeo-18 Lux first-in-man studies^9^^,^ 10 and currently available data from real world registries on the use of DCBs in femoropopliteal arteries which report 12-month fCD TLR up to 94%.^11^^-^ 13 The use of additional devices (atherectomy, scoring balloons, stents) was not restricted in BIOLUX P-III. Yet, despite 76.3% of lesions being calcified (and 11.7% heavily calcified), only 13.2% of the lesions required a stent, compared to about 25% in other registries.^12^^,^ 13 These results support use of Passeo-18 Lux as a stand-alone treatment for atherosclerotic infrainguinal lesions, thus reducing the need for durable implants even when lesions are calcified. Moreover, in a cohort of patients in which 40% of patients had CLI (including 30% with Rutherford class 5 or 6), the BIOLUX P-III all-comers data demonstrate statistically significant improvements in Rutherford classification and pain score, with 82.7% and 75.4% improvement at 12 months respectively.

In view of resource constraints and soaring health care costs, the need for cost-effective therapies is critical. In Brazil, for instance, health care costs associated with PAD endovascular revascularization increased 92% between 2008 and 2012.^14^ Several studies^15^^-^ 17 have suggested DCB’s cost-effectiveness for the treatment of atherosclerotic femoropopliteal arteries compared to PTA. However, each DCB is different and must be evaluated in randomized and real-world clinical investigations to demonstrate their performance and safety. Passeo-18 Lux DCB safety and effectiveness are supported by a robust set of clinical evidence comparable to other devices with published clinical data, as indicated by the 12-month CD TLR rate (6.8%) in particular. Real-world evidence has demonstrated consistent results and improvement in clinical outcomes such as Rutherford classification and pain scale.

### Study limitations

Lack of randomization and control group could be seen as limitations. However, unlike randomized studies, real-world studies are not restricted to low risk patients who are most likely to benefit from the treatment. To comply with standards of care, neither angiographic nor duplex ultrasound were mandated. Hence, the efficacy secondary endpoint, primary patency, could not be systematically assessed. Data published here are not complete and only concern the 12-month results of patients included during the first year of enrolment.

## CONCLUSION

In a real-world environment, the BIOLUX P-III All-Comers registry preliminary results confirm the safety and efficacy of Passeo-18 Lux DCB as a stand-alone treatment option for PAD in infrainguinal arteries. Those findings are encouraging and consistent with results deriving from first-in-man RCTs. Upcoming full cohort data, subgroups analysis (diabetes, female sex, CLI, infrapopliteal lesions) and longer-term, 24-month, follow-up data will enable determination of the benefit of Passeo-18 Lux in high-risk populations and complex lesions and how this benefit is sustained over time.
